# Development, Current Status, and Remaining Challenges for Respiratory Syncytial Virus Vaccines

**DOI:** 10.3390/vaccines13020097

**Published:** 2025-01-21

**Authors:** Cleo Anastassopoulou, Snežana Medić, Stefanos Ferous, Fotini Boufidou, Athanasios Tsakris

**Affiliations:** 1 Department of Microbiology, Medical School, National and Kapodistrian University of Athens, 11527 Athens, Greece; sferous@med.uoa.gr (S.F.); atsakris@gmail.com (A.T.); 2 Department of Epidemiology, Faculty of Medicine, University of Novi Sad, 21000 Novi Sad, Serbia; snezana.medic@mf.uns.ac.rs; 3 Center for Disease Control and Prevention, Institute of Public Health of Vojvodina, 21000 Novi Sad, Serbia; 4 Neurochemistry and Biological Markers Unit, 1st Department of Neurology, Eginition Hospital, Medical School, National and Kapodistrian University of Athens, 11528 Athens, Greece; fboufidou@med.uoa.gr

**Keywords:** respiratory syncytial virus (RSV), lower respiratory tract disease (LRTD), prefusion F vaccine, maternal immunization, older adults

## Abstract

Respiratory syncytial virus (RSV) causes significant morbidity and mortality, especially in young children and the elderly. RSV vaccine development puzzled vaccinologists for years. Safety concerns of initial formulations, the lack of an absolute correlate of protection, and the need for selecting appropriate virus attenuation and antigen–adjuvant combinations contributed to delayed vaccine production. The recent stabilization of the RSV-F glycoprotein in the prefusion (preF) conformation that constitutes the primary target of RSV-neutralizing antibodies was key for efficient vaccine design. Two protein subunit vaccines (GSK’s Arexvy and Pfizer’s Abrysvo) and one mRNA RSV vaccine (Moderna’s mRESVIA) are now available. This article aims to provide a comparative overview of the safety and efficacy of novel RSV vaccines that are approved for the prevention of RSV-lower respiratory tract disease (LRTD) in adults 60 years of age and older, with updated recommendations calling for the expansion of vaccination to all adults at increased risk for severe RSV disease. Abrysvo is the only vaccine indicated for use in pregnancy to prevent RSV-LRTD in infants from birth to 6 months of age. We provide a comparative assessment of the efficacy of approved RSV vaccines over a maximum of three seasons, summarizing currently available data. We conclude that despite the decreasing vaccine efficacy over time, which should be anticipated for a virus that is characterized by short-term immunity, efficacy was clinically meaningful over placebo. The increased risk of Guillain–Barré syndrome post vaccination with Abrysvo or Arexvy, which prompted the FDA to require the inclusion of such warnings in the prescribing information of these two RSV vaccines, should be prioritized and investigated thoroughly. Furthermore, ongoing vaccine surveillance and further evaluation, particularly among immunocompromised patients, frail elderly subjects, and young infants that were under- or not represented in pivotal clinical trials, are necessary. As in the success story of combined pediatric vaccines, combination vaccines, conferring protection against several respiratory illnesses in one dose, could help improve vaccine acceptance and coverage rates in older adults.

## 1. Introduction

The original name given to respiratory syncytial virus (RSV) when it was first discovered in 1955 was “Chimpanzee Coryza Agent” [1]. As reviewed by Ruckwardt, the virus was thereafter shown to commonly cause serious lower respiratory tract infections (LTRIs) in infants and young children [1]. Our knowledge of the biological and pathogenetic characteristics of RSV has expanded since then.

RSV is an RNA virus of the *Paramyxoviridae* family and *Pneumovirus* genus [2]. Ten functional genes (3′ NS1-NS2-N-P-M-SH-G-F-M2-L) are encoded by its single-stranded, non-segmented, negative-sense, approximately 15 kb RNA genome [2,3]. Each gene encodes one protein, except for M2 which contains two slightly overlapping open reading frames (ORFs) encoding for two distinct proteins, M2-1 and M2-2 [4]. The fusion (F) and attachment (G) glycoproteins protruding from the viral envelope are the primary targets of neutralizing antibodies [1]. The highest genetic diversity has been observed in G of the two known RSV subtypes, A and B [1,5]. Each RSV subtype contains multiple genotypes [1,5]. F, a class I viral fusion protein, is highly conserved genetically and antigenically [6]. During fusion, the F protein undergoes an irreversible transformation from a metastable to a highly stable conformation (preF to postF, respectively) [1]. Sites Ø and V on the apex of the metastable preF are targeted by the most potent neutralizing antibodies; thus, F-based vaccines displaying these antigenic sites while retaining the preF structure are suitable for RSV vaccine candidates [7,8,9,10].

Epidemiological studies estimated that, globally, RSV was responsible for 33 million acute LTRI, 3.6 million hospitalizations, and 101,400 deaths (26,300 in-hospital) in children less than 5 years of age in 2019 [11]. Most hospitalizations occur in infants aged less than 6 months, and most RSV-associated deaths in children aged under 5 years occur in low- and middle-income countries (LMICs) and in the community rather than hospitals [1,11,12]. Factors predisposing children to severe RSV disease include prematurity, chronic lung disease, and congenital heart disease [1]. Meanwhile, RSV infection in early life has been associated with childhood asthma and impaired lung function [1].

Significant morbidity and mortality are also caused by RSV in the elderly and immunocompromised, as shown by a recent meta-analysis in high-income countries in 2019 that attributed 470,000 hospitalizations and 33,000 in-hospital deaths in adults over the age of 60 globally to RSV [13]. The true RSV burden in older adults may be higher in reality. Adult viral titers are lower compared to those of infected children; hence, the typically used reverse transcription-polymerase chain reaction (RT-PCR) test from nasopharyngeal swabs of adult patients may result in under-diagnosis [14]. Employing a wider variety of samples, including saliva, serum, and sputum, especially from hospitalized adults suffering from acute respiratory infection (ARI), may ameliorate RSV diagnosis [1]. Long-term effects that could lead to the worsening of comorbid conditions are not infrequent among older adults with severe RSV disease during the acute phase of infection. Such long-term effects could render necessary the usage of additional healthcare services.

The disease peak in young infants despite the presence of maternally acquired antibodies, in conjunction with the multiple RSV infections that humans experience throughout life, foretells the biological difficulties in inducing protective immunity with a vaccine [1]. Indeed, efforts to develop an RSV vaccine were initially met with failure. A formalin-inactivated (FI-RSV) vaccine tested in infants in the 1960s resulted in an unacceptable rate of hospitalization of vaccinees, in addition to the deaths of two infants [15]. Although the exact mechanism responsible for this paradoxical susceptibility following vaccination is still unknown, it is theorized that the vaccine did not protect vaccinated individuals, due to a suboptimal induction of neutralizing antibody production [16]. Other groups investigated the possible deleterious effect of maternal antibodies against RSV and the blunting effect they may exert on the immune responses of infants [17]. This trial increased awareness for the immunological ambiguity of RSV and how a successful vaccine must not induce severe RSV disease, since RSV disease might be in part immune-mediated [18].

Tackling the conundrum of RSV vaccines that would require protecting both high-risk adults and young children and infants has continued to puzzle vaccinologists. To date, three vaccines have been approved and are currently in use in the so-called “developed” world. We have recently summarized the latest findings on available vaccines for the elderly, including RSV, and examined vaccine recommendation differences for this age group between countries of the European Union (EU)/European Economic Area (EEA) and the United States (US) [19]. The aim of the present narrative review was to provide a comparative overview of the safety and efficacy of the novel RSV vaccines. We also describe the milestones on the road to the newly approved RSV vaccines and their current licensure status in Europe and the US. Furthermore, we discuss the remaining challenges for the prevention of severe RSV infections in the pediatric, adult, and elderly populations.

## 2. Methodology

For writing this narrative review, the PubMed database was searched for English-language original articles or reviews in peer-reviewed journals up to early December 2024, using the medical subject heading (MeSH) terms “vaccine” and the viral pathogen in question, i.e., “respiratory syncytial virus (RSV)”. To supplement these MeSH terms, paraphrased or synonymous terms were also used, including, for example, “immunization” or “vaccination” instead of “vaccine” and the name of each novel vaccine, e.g., “RSVPreF3 (Arexvy)”. Reference is made to articles published in moderate- to high-impact Abridged Index Medicus (AIM) journals. Where possible, we focused on more recent articles as well as on the results of clinical trials. We provide a description of the difficulties associated with developing safe and effective RSV vaccines and the milestones on the road to the newly approved RSV vaccines. The latest vaccine recommendations for EU/EEA countries and the US were sought on the European Centre for Disease Prevention and Control (ECDC) and the US Centers for Disease Control and Prevention (CDC) databases and their publications, as well as in PubMed. Safety and efficacy results from clinical trials are presented in chronological order.

## 3. Epidemiology of RSV Infections

RSV is a frequent cause of mild and self-limited respiratory tract infections worldwide [20]. The true burden of RSV-associated ARIs remains largely underestimated, mostly due to undetected infections [14,21]. As already mentioned, the risk of a serious course of RSV infection is increased in premature children, newborns and young children, the immunocompromised, pregnant women, and the elderly [22,23]. Up to 70% of all childhood respiratory infections are caused by RSV, with the greatest burden in infants living in LMICs [21]. RSV-ARI remains one of the most common reasons for the hospitalization of otherwise healthy infants and young children in developed countries [24], but also the most common cause of bronchiolitis and pneumonia in infants globally [20]. Although 90% of children are already infected by the age of 2, reinfections in older age are common due to the short-lived immunity to RSV [22].

Transmissibility, as assessed by the basic reproduction number (R_0_), or secondary attack rate, puts RSV in the range of the most contagious respiratory viruses [25,26]. In a study by van Boven et al., the estimated R_0_ of RSV was generally high (R_0_ > 10), while sensitivity was higher in older adults (≥65 years) [26]. Transmission of the virus takes place when an infected person coughs or sneezes, through direct contact and recently contaminated surfaces [20,23]. An individual may be contagious up to 2 days before symptoms appear. The length of RSV shedding varies depending on the severity of the infection and the individual’s immune status, but usually lasts between three and eight days [20]. Significantly longer shedding, lasting for weeks, has been reported in immunocompromised patients [20].

The severity and timing of the RSV season in a community vary from year to year. RSV displays a seasonal pattern of infectivity similar to influenza, with annual recurrence [21]. A and B subtypes generally cocirculate, although one subtype may predominate during a season [1]. The RSV season usually peaks during the winter (December–January) in countries with a temperate climate [20,27]. Outbreaks in kindergartens and schools are common and often spread to families through infected children. The frequency and extent of outbreaks caused by RSV are highly dependent on the declining level of background immunity of the population [24,28]. In some scenarios, estimates of the rate of loss of natural immunity were around 6% per year in most age groups, but much higher in the elderly (~15% per year), implying a greater susceptibility to infection in older age groups [26]. Other factors, such as the birth rate, overcrowded living conditions, temperature, and humidity, also affect the seasonal peaks of RSV infections [24,27].

Viral interference and competition for the same host can greatly affect the circulation of seasonal and pandemic respiratory viruses in the community, as observed during the COVID-19 pandemic when all rates of viral respiratory infections, especially RSV-ARI and flu, were drastically reduced [29]. After the lifting of non-pharmaceutical measures, a rebound of RSV-ARIs, including unusual off-season outbreaks and a delayed increase in RSV compared to the usual seasonality, were registered in many countries [30,31]. The concept of “immunity debt” has been proposed and is widely accepted as an explanation for this atypical seasonal increase in RSV and other respiratory infections worldwide [32].

Those at the greatest risk for severe RSV infection are seniors over the age of 75, especially those with underlying medical conditions such as diabetes, chronic heart or lung disease, the immunocompromised, the severely obese, and those living in long-term care facilities, such as nursing homes [20]. Although a small percentage of adults infected with RSV require hospitalization, RSV-ARI remains one of the most common indications for hospital admissions in people aged ≥65 years [21]. In addition, RSV can sometimes lead to the worsening of chronic obstructive pulmonary disease (COPD), asthma, and congestive heart failure, especially in the elderly [20]. The community transmission of RSV in nursing homes and long-term facilities through contact with family members and caregivers is common. High-income countries are considered at particular risk of RSV outbreaks with the highest burden in the elderly due to population aging, immunosenescence, and the increased burden of comorbidities [33]. Despite the availability of vaccines and antiviral drugs, the focus of RSV prevention remains on non-pharmaceutical interventions, including frequent hand washing, covering the mouth and nose when sneezing or coughing, avoiding close contact with patients with RSV-ARI, and improving general and personal hygiene.

## 4. History of RSV Vaccine Development

Studies on the RSV vaccine began shortly after the discovery of the virus. The FI-RSV vaccine, the first RSV vaccine, developed in the 1960s, was highly reactogenic [16]. Four clinical trials conducted in 1966–1967 tested this inactivated RSV vaccine in immunologically naïve children without prior exposure to RSV. As reported in one of the studies, most of the vaccinated children were hospitalized after contracting wild-type RSV, including two vaccinated toddlers who died [16,34]. The cause of death was enhanced vaccine-associated respiratory disease (ERD) induced by antibody-dependent enhancement.

The mystery of FI-RSV vaccine failure was not solved until 2008, when Delgado and his team published the results of their experimental study in *Nature Medicine* [35]. The authors confirmed that inactivated RSV vaccines induce poor toll-like receptor (TLR) stimulation, a finding which was subsequently associated with a lack of maturation and production of nonprotective antibodies [35,36]. Numerous clinical trials followed, accompanied by studies in animal models, which together elucidated the various immunological processes associated with FI-RSV ERD syndrome [37]. They have demonstrated that infants vaccinated with FI-RSV had tissue infiltrates of inflammatory mononuclear cells in addition to eosinophils, neutrophils, and lymphocytes due to a robust CD4+ T cell immune response following RSV infection [37]. Furthermore, there was immunohistochemical evidence of immune complex deposition and complement fixation in tissues.

Lessons learned from the failure of the first RSV vaccines have shown that future RSV vaccines in immunologically RSV-antigen-naive infants should avoid inducing Th2-biased CD4+ T cell responses and binding antibodies with low neutralizing potential [37]. The ultimate goal of successful immunization in infants without RSV immunity would be to induce highly neutralizing antibodies along with supportive T helper responses, and sufficient numbers of CD8+ T cells to eliminate viral infection and reduce shedding [37].

The issue of vaccine safety in immunologically naïve children and the lack of an absolute correlate of protection against clinically relevant RSV infection have been the main challenges in RSV vaccine development [38]. RSV reinfections are frequent due to the short-term immunity after the initial infection, particularly in young infants [39]. As with SARS-CoV-2 and other viruses that infect mucosal surfaces without a viremic phase, repeated RSV infections are common and typically result in relatively short-lived antibody responses [40,41,42]. Virus transmission among siblings and within families, as well as repeated infections in the same person with either homologous or heterologous subtypes of RSV within the same or subsequent season, are often encountered [43]. Given that RSV vaccines were also intended for the elderly, it was necessary to select vaccines with the appropriate dose and combination of antigens and adjuvants to enhance the immune response generated by the vaccine [44].

Dozens of other failed attempts have long stalled RSV vaccine progress. Over the next two decades, researchers developed several live attenuated vaccines with good safety profiles, but which were not shown to be immunogenic enough to provide effective protection against RSV in all age groups, due to the short “therapeutic window”. Namely, it has been established that a live RSV vaccine that is sufficiently attenuated for RSV-naive children can be excessively attenuated for older children and adults, which is why these vaccines were poorly immunogenic in these age groups despite displaying a good safety profile [39,45,46]. Safety and efficacy in the RSV-seronegative target population remained unknown because vaccine candidates were only tested pre-clinically or in RSV-seropositive people. Ultimately, after many attempts, scientists concluded that RSV vaccine candidates had failed because none produced effective neutralizing antibodies [47].

It was only experimental studies in mice by McLellan and Graham that led to the discovery of an antibody that effectively neutralized RSV’s preF protein without binding to the postF protein [48]. This antibody was about 50 times more potent than the murine precursor of Palivizumab (Synagis^®^), a humanized monoclonal antibody preparation intended for prophylaxis against serious RSV illness in high-risk infants [49]. Over the next few years, scientists grew human cells to produce the prefusion protein and tried to purify it for RSV vaccine production. In the period of 2017–2019, many clinical trials were conducted to evaluate the safety of the RSV vaccine candidates, including particle-based, attenuated, or chimeric, subunit and vector-based RSV vaccines [38,44].

## 5. Approval Status, Safety, and Efficacy of Approved RSV Vaccines

Approved RSV vaccines by regulatory authorities in the EU/EEA and the US were constructed based on two types of vaccine technologies: protein subunit and mRNA-based platforms. Two protein subunit RSV vaccines, RSVPreF3 (Arexvy, GSK) and RSVPreF (Abrysvo, Pfizer), and one mRNA RSV vaccine, mRNA-1345 (mRESVIA, Moderna), are available at present. The current approval status of RSV vaccines is presented in Table 1.

Following the updated (as of 26 June 2024) recommendations by the US Advisory Committee on Immunization Practices (ACIP), a single dose of any FDA-approved RSV vaccine (Arexvy (GSK), Abrysvo (Pfizer), or mRESVIA (Moderna)) is recommended for all adults aged ≥75 years and for adults aged 60–74 years who are at increased risk for severe RSV disease [50]. Another dose is not recommended for adults who have previously received an RSV vaccine [50]. The results of the principal safety and efficacy of clinical trials of these vaccines are presented in Section 5.1 and Section 5.2 that follow. Table 2 summarizes the reported vaccine efficacy of the three approved vaccines against RSV-associated LRTD in older adults. Definitions of endpoints and of what constitutes LRTD and severe LRTD differ between the studies, rendering direct comparisons challenging.

### 5.1. Protein Subunit RSV Vaccines

#### 5.1.1. RSVPreF3 (Arexvy, GSK)

The first RSV vaccine, RSVPreF3 (Arexvy, GlaxoSmithKline Biologicals [GSK]), was approved by the FDA for the prevention of ARI in subjects aged over 60 years on 23 May 2023 and by the EMA in June 2023 [56,57]. Arexvy is a recombinant, single-dose vaccine that contains a liposomal formulation of pre-F RSV-A together with Adjuvant System 01_E_ (AS01_E_) (Table 1). AS01_E_ contains 3-O-desacyl-4′-monophosphoryl lipid A (MPL) from *Salmonella Minnesota* and the plant extract *Quillaja saponaria* Molina, fraction 21 (QS-21) [57]. Adults aged 50–59 at increased risk for severe RSV disease, with comorbid conditions such as chronic cardiovascular disease, chronic lung or respiratory disease, or diabetes with complications, are now included in the age indications for Arexvy (Table 1).

Three RSVPreF3 vaccine formulations (30, 60, and 120 μg) were tested in an initial phase I/II clinical trial in two population groups: without the adjuvant in young adults (18–40 years of age), and both without and with the adjuvant in older adults (60–80 years of age) [58]. Higher reactogenicity was found in adjuvanted vaccines, but with no major safety concerns since solicited adverse events tended to be mild to moderate and transient [58]. Nevertheless, higher-than-expected background rates (1.8 cases per million administered Arexvy doses) of Guillain–Barré syndrome (GBS) were reported to pharmacovigilance databases, i.e., to V-safe and to the Vaccine Adverse Event Reporting System (VAERS), 42 days post-RSV vaccination [59]. The potential association of GBS with RSV vaccination is under scrutiny by regulatory authorities.

As reported in the phase I/II clinical trial by Leroux-Roels et al. [58], more robust cellular immune responses, as assessed by intracellular cytokine staining on peripheral blood mononuclear cells, were induced in older adults vaccinated with the vaccine containing the adjuvant compared to unadjuvanted formulations. The boosting of humoral (RSVPreF3-specific immunoglobulin G [IgG] and RSV-A-neutralizing antibody) and cell-mediated immune responses in older adults, in conjunction with the acceptable safety profile, favored the selection of the 120 μg AS01E formulation for further development as a single-dose vaccine [58]. It should be noted that robust immune responses were induced against both RSV subtypes, even though the vaccine contained the RSV-A preF protein [58].

Efficacies of 71.7% (95% CI, 56.2 to 82.3) against RSV-ARI, 82.6% against RSV-LRTD, and 94.1% against severe RSV-LRTD, defined by the presence of at least two lower-respiratory-tract clinical findings or by the need for non-invasive or invasive oxygenation, were reported in AReSVi-006, the phase III pivotal clinical trial of Arexvy in adults aged over 60 years (Table 2) [55]. During one RSV season (over a median follow-up of 6.7 months), vaccine efficacy, against both RSV-A and RSV-B, was 94.6% in older adults with at least one underlying relevant medical condition [55]. The severity of RSV-associated symptoms was also attenuated in breakthrough infections, with trends of a reduced impact on physical functioning and the health utility of older adults [60]. Injection site pain, headache, fatigue, and myalgia, as well as immune-mediated involvement of the musculoskeletal and connective tissue and of the respiratory and nervous systems, were among the most frequently reported adverse events, with an equal, nonetheless, distribution of serious unsolicited reactions among placebo and vaccine recipients [55].

Across two RSV seasons (over a median of 17.8 months), efficacy rates of 67.2% (97.5% CI, 48.2–80.0%) against RSV-LRTD and of 78.8% (95% CI, 52.6–92.0%) against severe RSV-LRTD were noted [61]. Overall vaccine efficacy was not increased by revaccination with a second, well-tolerated vaccine dose prior to the second RSV season, 67.1% (97.5% CI, 48.1–80.0%) against RSV-LRTD, and 78.8% (95% CI, 52.5–92.0%) against severe RSV-LRTD [61].

New data from the ongoing AReSVi-006 phase III trial that were announced in early October 2024 by GSK indicated that a single dose of Arexvy was protective across three seasons (30.6 months of median follow-up) and, thus, against different RSV subtypes, even in adults aged 70 to 79 years old and in patients with underlying medical conditions [51]. The reported cumulative efficacy rates after three RSV seasons were 62.9% against RSV-caused LRTD and 67.4% against severe disease when compared with placebo (Table 2), suggesting that patients may be vaccinated against RSV all year round [51]. In agreement with pre-licensure clinical trials and surveillance systems such as VAERS, follow-up data from three RSV seasons that were recently presented at the ACIP meeting also confirmed, nonetheless, the potentially increased risk for GBS following vaccination with Arexvy among adults aged 65 years and older [62].

Arexvy is not indicated for pregnant people. Safety concerns led to an early cessation of a phase III trial of Arexvy in 5328 pregnant women and 5233 infants from 24 countries [63].

A higher proportion of women in the vaccine group delivered prematurely (6.8% in the vaccine group vs. 4.9% in the placebo group, relative risk 1.37; 95% CI, 1.08–1.74; *p* = 0.01), for reasons that remain unexplained [63]. In 13 of 3494 (0.4%) and 3 of 1739 (0.2%) respective cases, deaths of neonates followed (relative risk, 2.16; 95% CI, 0.62–7.56; *p* = 0.23) [63].

#### 5.1.2. RSVPreF (Abrysvo, Pfizer)

The second RSV vaccine, RSVpreF (Abrysvo, Pfizer), was approved by the FDA on 31 May 2023 for the prevention of RSV-LRTD in adults aged ≥60 years [64], and on 21 August 2023 for pregnant subjects for the passive protection of infants from birth to 6 months of age against RSV-LRTD and severe RSV-LRTD [64]. Abrysvo was also approved by the EMA for the same indications, with maternal immunization allowed after the 24th week of gestation [65]. Expansion of the FDA-recommended age limits for vaccination to both older and younger spans followed, while Abrysvo remains the only RSV vaccine indicated for maternal immunization (Table 1). Equal amounts of pre-F RSV-A and RSV-B antigens and no adjuvant are contained in Pfizer’s Abrysvo recombinant protein subunit vaccine (Table 1).

A phase I/II study that evaluated the safety and immunogenicity of different concentrations of the vaccine (60, 120, or 240 µg) in the presence or absence of Al(OH)_3_ adjuvants in adults aged 18–49 (N = 618) demonstrated that the vaccine was safe [66]. Mild or moderate local (mostly pain at the injection site) and systemic side-effects, such as headache, fatigue, and myalgia, were reported, but with no association with the presence of adjuvants or with any particular vaccine dose [66]. Induced neutralizing antibody titers, which were compared to neutralizing titers of Palivizumab at a serum level of 100 μg/mL, essentially defined immunogenicity [66]. Robust neutralizing antibody titers, higher in women compared to men—a promising finding for the protection of neonates via maternal immunization—were produced and maintained above baseline for about a year post vaccination [66].

A phase I/II study in healthy adults aged 65–85 years reported similar results, without further enhancement in humoral and cellular responses by the oligodeoxynucleotide adjuvant CpG [67]. Subsequent clinical development proceeded with the unadjuvanted 120 µg dose level of RSVpreF. This vaccine dose proved to be safe and effective against symptomatic RSV infection and viral shedding, in a challenge study with RSV-A Memphis 37b [68].

As indicated in the interim analysis of a phase III clinical trial in adults aged ≥60 years, vaccine efficacy was 66.7% (96.66% CI, 28.8–85.8) against RSV-associated LRTD with at least two signs or symptoms and 62.1% (95% CI, 37.1–77.9) against RSV-associated ARI, after one RSV season [69]. The final results of this analysis that were published in late October 2024 [52] are presented in the summary in Table 2 in comparison with the other two RSV vaccines. This overview of the available data indicates that, although it is not currently recommended, revaccination may be needed for protection against RSV-LRTD in the seasons to come, independently of which RSV vaccine is used [52].

The findings of another recently published study expand upon pre-licensure trial results [55,69], by providing evidence of vaccine protection against RSV-associated hospitalization among US adults aged 60 years and older, including adults aged 75 years and older and those with immunocompromising conditions [70]. Nevertheless, recent real-world data from V-safe and the VAERS indicate that the Guillain–Barré (GBS) incidence following vaccination with RSVpreF might be higher than the estimated expected background rates in a vaccinated population [59]. The GBS reports were 4.4 cases per million administered doses of Abrysvo [59].

Regarding maternal immunization, phase IIb trials indicated that the unadjuvanted 120 mg vaccine formulation induced robust antibody responses in women, with follow-up measurements of anti-RSV neutralizing antibody titers both in umbilical cord and in transplacental blood samples [71]. Adverse events were more common in mothers who received adjuvanted vaccine formulations [71]. The phase III trial, which investigated the efficacy of Abrysvo in preventing medically attended RSV LRTD in infants following the vaccination of mothers, showed an efficacy of 81.8% within 90 days after birth and 69.4% within 180 days after birth [72]. Interim data did not indicate a statistically significant reduction in non-severe RSV infections 90 days after birth. However, a reduction in RSV infections 360 days after birth and in RSV-related hospitalizations 180 days after birth was noted [72]. Although the trial enrolled pregnant women from 24 to 36 weeks of gestation, vaccine efficacy appeared to be higher when the vaccine was administered between weeks 32 and 36 [73]. Premature births were noted in both phase II and phase III trials, with 5.3% in the vaccination group in the phase II trial compared to 2.6% in the placebo group and 5.7% in the vaccination group in the phase III trial compared to 4.7% in the placebo group [73]. It has yet to be established whether there is a causal relationship between vaccination and preterm birth or Guillain–Barré syndrome.

### 5.2. mRNA RSV Vaccines

#### mRNA-1345 (mRESVIA, Moderna)

Approved by the FDA in May 2024 and by the EMA in August 2024, Moderna’s mRNA-1345 (mRESVIA) is the most recently approved RSV vaccine. It is an mRNA-based vaccine encoding the membrane-anchored RSV-A preF encapsulated in a lipid nanoparticle (Table 1) [74]. mRESVIA, the second mRNA vaccine produced by Moderna after mRNA-1273 (Spikevax), is indicated for the prevention of RSV-LRTD in adults aged over 60 years (Table 1) [74].

The results from the ConquerRSV clinical trial, which were published in the *New England Journal of Medicine* in 2023, formed the basis for the regulatory authorities’ decision to approve mRESVIA [75]. A previous phase I clinical trial had not identified any safety concerns [76]. Vaccine efficacies of 83.7% (95.88% CI, 66.0 to 92.2) and 82.4% (96.36% CI, 34.8 to 95.3) against RSV-LRTD as defined by at least two and three symptoms, respectively, were found by the ConquerRSV study [75]. Only mild to moderate and transient adverse reactions were reported [75]. The most common systemic adverse reactions were fatigue, headache, myalgia, and arthralgia, while pain at the injection site was the most frequently reported local adverse reaction [75]. In contrast to mRNA vaccines against SARS-CoV-2/COVID-19 that are known to be related to the occurrence of myocarditis principally in younger male subjects [77], no reports of other adverse reactions, such as GBS or acute disseminated encephalomyelitis, were recorded [75].

Following a subsequent phase I dose-ranging study in adults aged 65 to 79 years [78], the 50 μg mRNA-1345 dose was selected for a phase II/III pivotal trial in such an immunosenescent population on the basis of its favorable profile of acceptable safety and tolerability and persistent immunogenicity for at least 12 months [78]. A booster vaccine dose did not lead to further elevated titers compared to the peak reached by the initial vaccination [78].

As in the ConquerRSV trial [75], enhanced nAb and bAb RSV responses were found, and, importantly, these responses were similar in subjects at risk of severe RSV disease, such as patients with relevant comorbid medical conditions and frail, older individuals [79]. Conferred protection waned over time, as evidenced, for example, by the decreasing efficacy in preventing LRTD with at least two symptoms from 78.7% (95.04% CI, 62.8–87.9), during a median 3.7-month follow-up, to 62.5% (95.04% CI, 47.7–73.1) at 8.6 months, and to 50.3% (95.04% CI, 37.5–60.7) at 18 months (Table 2). Still, mRNA-1345 remained efficacious at preventing severe RSV disease, including among high-risk individuals [53]. Revaccination with mRNA-1345 to boost waning humoral and cellular immune responses and enhance protection, particularly for older adults, may be recommended at specified intervals, possibly annually, in the future.

## 6. Recommendations for RSV Vaccination: Timing, Number of Doses, and Coadministration with Other Vaccines

Irrespective of the vaccine type and manufacturer, current recommendations indicate that RSV vaccines should be given as a single dose, preferably prior to the start of the RSV season (typically in late autumn until winter) [80]. Eligible adults (as specified in Table 1) do not need to receive a dose every RSV season, since the RSV vaccine is not currently an annual vaccine [80]. Thus, additional doses are not recommended for adults who have previously received an RSV vaccine [50].

Protein subunit RSV vaccines can be administered concomitantly with seasonal influenza vaccines (standard dose unadjuvanted, high-dose unadjuvanted, or standard dose adjuvanted) [57,65]. Numerically lower RSV-A and -B neutralizing titers and numerically lower influenza A and B hemagglutination inhibition titers were observed upon concomitant administration of Arexvy or Abrysvo with seasonal influenza vaccines, as compared to the separate administration [57,65]. These findings were not observed consistently across studies and their clinical relevance is unknown [57,65].

GSK’s Arexvy can be administered simultaneously with any of the following vaccines: COVID-19 vaccines, recombinant zoster vaccine, pneumococcal vaccines, and Td/Tdap vaccines [57]. However, in the case of concomitant administration with another injectable vaccine, the vaccines should always be administered at different injection sites.

Pfizer’s Abrysvo may be administered with a recommended minimum interval of two weeks of administration of a tetanus, diphtheria, and acellular pertussis vaccine (Tdap) [65]. No safety concerns were identified in healthy non-pregnant women when Abrysvo was co-administered with Tdap. Although immune responses to RSV-A, RSV-B, diphtheria, and tetanus were non-inferior on co-administration compared to separate administration, the immune responses to the pertussis components were lower and non-inferiority criteria were not met [65].

Available data on the reactogenicity and immunogenicity of coadministration of RSV vaccines and other vaccines are limited at present, and no such data are yet available for mRESVIA [54].

## 7. Remaining Challenges for RSV Vaccines

Real-world data on the efficacy and duration of protection of the newly approved vaccines against severe RSV disease in high-risk older adults and infants are still lacking. Post-marketing surveillance data for potential adverse events following RSV vaccine administration are also lacking. Most available data are derived from the pre-clinical and pivotal clinical trials that are discussed in Section 4. Further observational data are certainly necessary. However, it should be noted that the direct comparison of available vaccines, primarily οf the two protein subunit vaccines, are difficult to interpret since the endpoints of the respective clinical trials differed in their definition of severe RSV LRTD. Moderna’s mRESVIA is based on a different (mRNA) vaccine platform, further complicating direct comparisons.

In addition, studies on specific subpopulations of interest are also lacking. For example, although the recently published ACIP recommendations for RSV vaccination include all older adults aged over 75, with vaccination for adults aged 60–74 years of age recommended for high-risk individuals (Table 1), those with severe immunocompromised states, such as active cancer undergoing chemotherapy or those receiving immunomodulatory therapies in the context of rheumatologic disease, were excluded from the pivotal clinical trials of all three vaccines.

To our knowledge, there are also little available data on the immunogenicity of novel RSV vaccines in immunocompromised individuals, with one available study investigating the immunogenicity of Abrysvo in immunocompromised individuals. Although the data have yet to be published for peer review, initial reports indicate that neutralizing responses were similar to those of immunocompetent individuals [81]. This is a pending issue that merits specific attention considering that many immunomodulatory therapies, particularly those associated with B-cell depletion (e.g., Rituximab), have been associated with decreased vaccine efficacy [82]. Therefore, it would be erroneous to extrapolate vaccine safety and efficacy data from immunocompetent individuals to immunodeficient patients. Similarly, frail individuals were not included in the Abrysvo, Arexvy, and mRESVIA trials and individuals over the age of 80 were insufficiently represented; thus, data on the efficacy of vaccination in groups that have the highest risk for severe RSV disease are still lacking.

Further complicating matters is the fact that the pivotal clinical trials for all vaccines were conducted during the COVID-19 pandemic while travel and social restrictions were in effect (2021 and 2022), which has apparently affected community RSV circulation [83]. Therefore, COVID-19 restrictions could have influenced reported vaccine efficacy.

Moreover, the RSV vaccination and management landscape, especially for infants, is not clear. Palivizumab and the recently approved Nirsevimab are the monoclonal antibodies currently available as prevention therapies of choice for infants. The use of Palivizumab is recommended at present in premature newborn infants (born at 29 weeks of gestation or earlier) and in high-risk infants such as those with significant congenital heart disease, immunocompromised conditions, or chronic lung conditions such as cystic fibrosis [84]. A long-acting monoclonal antibody, Nirsevimab, is indicated for RSV prophylaxis in all infants younger than 8 months during their first RSV season, with a subsequent second dose for all infants younger than 20 months who are considered high-risk for severe RSV infection during their second RSV season [85]. The lower cost [86], greater duration of protection, and ease of use (since it requires one dose compared to the once-a-month dose required by Palivizumab) render Nirsevimab probably preferable to Palivizumab.

The CDC recommends either Abrysvo or Nirsevimab as equally effective [87]. Disadvantages of Abrysvo compared to Nirsevimab include (i) the as-of-yet unknown duration of protection in infants, with at least data from initial clinical trials demonstrating a reduction in protection after a few months, and (ii) the variant seasonality of RSV across different geographical regions which affects the timing of vaccine administration to the mother, and which therefore might reduce compliance and the overall efficacy of vaccine delivery [88]. Furthermore, there is a consistent undercoverage of pregnant women for other vaccine-preventable diseases such as tetanus, diphtheria, pertussis, influenza, and COVID-19 [89], which might extend to RSV vaccinations as well. Observational reports have also indicated that vaccination is not a priority for obstetricians, which could further result in low vaccine uptake from pregnant mothers [90]. Conversely, Nirsevimab is more expensive. Mathematical projection models have indicated that both are similarly cost-effective, since Abrysvo might reduce the maternal RSV disease burden even with a lower projected coverage rate than Nirsevimab [91].

Finally, it is still uncertain whether mothers already vaccinated once with Abrysvo ought to be revaccinated in subsequent pregnancies or whether maternal comorbidities, such as immunosuppression, might affect vaccine efficacy and infant protection, since most mothers with chronic conditions were excluded from the phase III Abrysvo trial.

## 8. Concluding Remarks

The recently approved vaccines against RSV are expected to be effective in reducing RSV disease burden in older adults and in infants in the future. Due to their recent approval, data regarding their efficacy, particularly in specific subpopulations not represented in pivotal clinical trials, are lacking. Further clinical and observational data are needed, especially in immunocompromised adults and mothers.

Considering the existence of expensive monoclonal antibody preventative therapies, the geographical variance of RSV seasonality and incidence, and low maternal vaccination rates, local and national healthcare services must tailor guidelines accordingly. Further data are also needed to augment current healthcare practices and delineate the role of monoclonal antibodies and vaccination in relation to RSV disease prevention in infants.

In addition, education of the public and the medical community is necessary to improve acceptance of the RSV vaccines. There is currently a wide gap in RSV and influenza vaccine coverage, although, in some seasons, RSV incidence and mortality exceed those of influenza.

Seniors are among the most vulnerable patient populations when it comes to infections from respiratory viruses, so the potential to immunize against multiple respiratory viruses with a single vaccine could increase vaccine uptake and immunity in the population. Combination vaccines have been a success for pediatric populations for over 70 years [92]. Still, their efficacy in the elderly has yet to be proven. Pending the licensing of combined polyvalent respiratory vaccines against RSV, influenza, and COVID-19 in the elderly, simultaneous vaccination may be beneficial and can help increase vaccine uptake and improve coverage and compliance with vaccine recommendations. In general, research into combination vaccines to facilitate acceptability and feasibility and ultimately increase vaccination rates in the adult population as well as across the entire age range is a future priority.

## Figures and Tables

**Table 1:** Current (as of November 2024) approval status of RSV vaccines for the prevention of RSV-LRTD in Europe and the United States. According to the updated recommendations by the US Advisory Committee on Immunization Practices (ACIP), a single dose of any FDA-approved RSV vaccine is recommended for all adults aged ≥75 years and for adults aged 60–74 years who are at increased risk of severe RSV disease, while no additional doses are recommended for adults who have previously received an RSV vaccine [50].

Vaccine (Manufacturer)	Vaccine Type (Active Substance)	Europe (EMA Indications)	United States (FDA Indications)
RSVPreF3 (Arexvy) (GSK)	Protein subunit (120 μg of RSV-A PreF3 Ag adjuvanted with AS01_E_ ^)	Adults aged 50–59 years at increased risk of RSV disease * All adults aged ≥60 years	Adults aged 50–59 years at increased risk of RSV disease * All adults aged ≥60 years
RSVPreF (Abrysvo) (Pfizer)	Protein subunit (60 μg of RSV-A PreF Ag and 60 μg of RSV-B PreF Ag)	All adults aged ≥60 years Pregnant individuals at 24–36 weeks of gestation to protect infants from birth up to 6 months	Adults aged 18–59 years at increased risk of severe disease * All adults aged ≥60 years Pregnant individuals at 32–36 weeks of gestation to protect infants from birth up to 6 months
mRNA-1345 (mRESVIA) (Moderna)	mRNA (50 μg of single-stranded 5′-capped mRNA encoding the RSV-A glycoprotein F stabilized in the prefusion conformation)	All adults aged ≥60 years	All adults aged ≥60 years

^ Adjuvant system AS01_E_ contains the plant extract *Quillaja saponaria* Molina, fraction 21 (QS-21) and 3-O-desacyl-4′-monophosphoryl lipid A (MPL) from *Salmonella Minnesota.* In GSK’s Arexvy vaccine, it is combined with RSV-A PreF3 Ag in a liposomal formulation. * Risk factors for severe RSV disease include the following conditions: chronic cardiovascular disease, chronic lung or respiratory disease, diabetes with complications, dependence on dialysis or end-stage renal disease, liver disease, hematologic conditions, severe obesity (body mass index ≥ 40 kg/m^2^), nursing home residence, and moderate or severe immune compromise. Abbreviations: EMA, European Medicines Agency; FDA, Food and Drug Administration; RSV, respiratory syncytial virus; RSV-LRTD, respiratory-syncytial-virus-associated lower respiratory tract disease.

**Table 2:** Efficacy of the three approved vaccines against RSV-associated LRTD in older adults.

	Vaccine Efficacy (%, CI, Cases in Vaccine Arm/Cases in Placebo Arm)		
Endpoint	RSVPreF3	RSVPreF	mRNA-1345
**Season 1**			
RSV-LRTD *	**82.6%** (96.95% CI, 57.9–94.1) 7/12,466 vs. 40/12,494 [51]	N/A	N/A
RSV-LRTD with ≥2 symptoms	N/A	**65.1%** (95% CI, 35.9–82.0) 15/18,050 vs. 43/18,074 [52]	**78.7%** (95.04% CI, 62.8–87.9) 15/17,561 vs. 70/17,503 [53]
RSV-LRTD with ≥3 symptoms	N/A	**88.9%** (95% CI, 53.7–?) ^ 2/18,050 vs. 18/18,074 [52]	**80.9%** (95.1% CI, 50.1–92.7) 5/17,561 vs. 26/17,503 [53]
Severe RSV-LRTD	**94.1%** ^†^ (95% CI, 62.4–99.9) 1/12,466 vs. 17/12,494 [51]	N/A	**86.7%** ** (95% CI, 41.9–97.0) [53]
RSV-LRTD in participants with ≥1 pre-existing comorbidity of interest	**94.6%** (95% CI, 65.9–99.9) 1/4937 vs. 18/4861 [51]	N/A	N/A
**Season 2**			
RSV-LRTD *	**56.1%** (95% CI, 28.2–74.4) 20/4991 vs. 91/10,031 [51]	N/A	N/A
RSV-LRTD with ≥2 symptoms	N/A	**55.7%** (95% CI, 34.7–70.4) 39/16,164 vs. 88/16,059 [52]	**62.5%** (95.04% CI, 47.7–73.1) 48/18,074 vs. 127/18,010 [53]
RSV-LRTID with ≥3 symptoms	N/A	**77.8%** (95% CI, 51.4–91.1) 8/16,164 vs. 36/16,059 [52]	**61.1%** (95.1% CI, 34.7–76.8) 20/18,074 vs. 51/18,010 [53]
Severe RSV-LRTD	**64.2%** ^†^ (95% CI, 6.19–89.2) 5/4991 vs. 28/10,031 [51]	N/A	**74.6%** ** (95% CI, 50.7–86.9) [53]
RSV-LRTD in participants with ≥1 pre-existing comorbidity of interest	**51.5%** (95% CI, 7.4–76.6) 12/1981 vs. 48/3895 [51]	N/A	N/A
**Season 3**			
RSV-LRTD *	**48.0%** (95% CI, 8.7–72.0) 16/4988 vs. 61/10,031 [51]	N/A	N/A
RSV-LRTD with ≥2 symptoms	N/A	N/A	**50.3%** (95.04% CI, 37.5–60.7) 113/18,181 vs. 225/18,132 [53]
RSV-LRTD with ≥3 symptoms	N/A	N/A	**49.9%** (95.1% CI, 27.8–65.6) 46/18,181 vs. 91/18,132 [53]
Severe RSV-LRTD	**43.3%** ^†^ (95% CI, −45.3–81.3) 6/4988 vs. 21/10,031 [51]	N/A	**56.7%** ** (95% CI, 33.1–72.6) [53]
RSV-LRTD in participants with ≥1 pre-existing comorbidity of interest	**57.8%** (95% CI, 8.0–83.0) 8/2000 vs. 37/3924 [51]	N/A	N/A
**Cumulative**	**Over 3 seasons** with season as covariate		
RSV-LRTD *	**62.9%** (97.5% CI, 46.7–74.8) 48/12,468 vs. 215/12,498 [51]	N/A	N/A
RSV-LRTD with ≥2 symptoms	N/A	N/A	**63.3%** (up to 12 months) (95% CI, 48.7–73.7) 47/18,112 vs. 127/18,045 [54]
RSV-LRTD with ≥3 symptoms	N/A	N/A	**63.0%** (up to 12 months) (95% CI, 37.3–78.2) 19/18,112 vs. 51/18,045 [54]
Severe RSV-LRTD	**67.4%** ^†^ (95% CI, 42.4–82.7) 15/12,468 vs. 75/12,498 [51]	N/A	N/A
RSV-LRTD in participants with ≥1 pre-existing comorbidity of interest	**64.7%** (95% CI, 45.1–78.1) 25/5014 vs. 116/4951 [51]	N/A	**63.4%** (up to 24 months) (95% CI, 45.4–75.5) 33/5393 vs. 88/5276 [53]

* Defined as ≥2 lower respiratory symptoms or signs (including ≥1 lower respiratory sign for ≥24 h), or ≥3 lower respiratory symptoms lasting for ≥24 h [55]. ^ Walsh et al. reported an upper 95% CI of 8.7, but we suspect that this may be a typo [52]. ^†^ Severe RSV-LRTD was determined on the basis of either of two case definitions: on the basis of clinical signs or investigator assessment or on the basis of receipt of supportive therapy [55]. ** RSV-LRTD-associated shortness of breath. Abbreviations: CI: confidence interval; N/A: not available; RSV, respiratory syncytial virus; RSV-LRTD: respiratory-syncytial-virus-associated lower respiratory tract disease.

## Data Availability

Not applicable.
