# Correction: Anastassopoulou et al. Development, Current Status, and Remaining Challenges for Respiratory Syncytial Virus Vaccines. *Vaccines* 2025, *13*, 97

**DOI:** 10.3390/vaccines13040354

**Published:** 2025-03-27

**Authors:** Cleo Anastassopoulou, Snežana Medić, Stefanos Ferous, Fotini Boufidou, Athanasios Tsakris

**Affiliations:** 1 Department of Microbiology, Medical School, National and Kapodistrian University of Athens, 11527 Athens, Greece; sferous@med.uoa.gr (S.F.); atsakris@gmail.com (A.T.); 2 Department of Epidemiology, Faculty of Medicine, University of Novi Sad, 21000 Novi Sad, Serbia; snezana.medic@mf.uns.ac.rs; 3 Center for Disease Control and Prevention, Institute of Public Health of Vojvodina, 21000 Novi Sad, Serbia; 4 Neurochemistry and Biological Markers Unit, 1st Department of Neurology, Eginition Hospital, Medical School, National and Kapodistrian University of Athens, 11528 Athens, Greece; fboufidou@med.uoa.gr

The authors would like to make the following corrections to this published paper [1].

In the original publication, there was an inaccuracy in Table 1 as the CDC instead of the FDA indications had been reported erroneously for RSVPreF in the United States. The corrected Table 1 is attached below.

Corrections were also necessary in Table 2 since the way we had originally presented vaccine efficacy gave the impression that the data are directly comparable. As already mentioned in the last sentence of the introduction of Section 5 (Approval Status, Safety, and Efficacy of Approved RSV Vaccines) and in the first paragraph of Section 7 (Remaining Challenges for RSV Vaccines) of the original publication, the definitions of the endpoints of the three RSV vaccines differ, and comparisons are difficult to interpret [1]. The edited Table 2 is attached below.

Consequent to these changes, the last two sentences of the fourth paragraph in Section 5.1.2, “RSVPreF (Abrysvo, Pfizer)”, should be replaced with the following: “This overview of the available data indicates that, although it is not currently recommended, revaccination may be needed for protection against RSV-LRTD in the seasons to come, independently of which RSV vaccine is used [52].”

The last sentence of the fifth paragraph in Section 5.1.2 of the original publication should be changed simply to “the GBS reports were 4.4 cases per million administered doses of Abrysvo [59]” because the increased incidence of GBS following RSVPreF was not statistically significant.

Finally, the Conflicts of Interest should be changed to “Prof. Tsakris participated once in an Advisory Board meeting on RSV prevention in adults held in Athens by Pfizer in 2023. All the other authors declare no conflicts of interest.” This information/potential minor conflict of interest was inadvertently omitted in the original version of the article.

With this correction, the order of some references has been adjusted accordingly.

The authors would like to apologize for any inconvenience caused to the readers by these changes. The authors state that the scientific conclusions are unaffected. This correction was approved by the Academic Editor. The original publication has also been updated.

## Figures and Tables

**Table 1:** Current (as of November 2024) approval status of RSV vaccines for the prevention of RSV-LRTD in Europe and the United States. According to the updated recommendations by the US Advisory Committee on Immunization Practices (ACIP), a single dose of any FDA-approved RSV vaccine is recommended for all adults aged ≥75 years and for adults aged 60–74 years who are at increased risk of severe RSV disease, while no additional doses are recommended for adults who have previously received an RSV vaccine [50].

Vaccine (Manufacturer)	Vaccine Type (Active Substance)	Europe (EMA Indications)	United States (FDA Indications)
RSVPreF3 (Arexvy) (GSK)	Protein subunit (120 μg of RSV-A PreF3 Ag adjuvanted with AS01_E_ ^)	Adults aged 50–59 years at increased risk of RSV disease * All adults aged ≥60 years	Adults aged 50–59 years at increased risk of RSV disease * All adults aged ≥60 years
RSVPreF (Abrysvo) (Pfizer)	Protein subunit (60 μg of RSV-A PreF Ag and 60 μg of RSV-B PreF Ag)	All adults aged ≥60 years Pregnant individuals at 24–36 weeks of gestation to protect infants from birth up to 6 months	Adults aged 18–59 years at increased risk of severe disease * All adults aged ≥60 years Pregnant individuals at 32–36 weeks of gestation to protect infants from birth up to 6 months
mRNA-1345 (mRESVIA) (Moderna)	mRNA (50 μg of single-stranded 5′-capped mRNA encoding the RSV-A glycoprotein F stabilized in the prefusion conformation)	All adults aged ≥60 years	All adults aged ≥60 years

^ Adjuvant system AS01_E_ contains the plant extract *Quillaja saponaria* Molina, fraction 21 (QS-21) and 3-O-desacyl-4′-monophosphoryl lipid A (MPL) from *Salmonella Minnesota.* In GSK’s Arexvy vaccine, it is combined with RSV-A PreF3 Ag in a liposomal formulation. * Risk factors for severe RSV disease include the following conditions: chronic cardiovascular disease, chronic lung or respiratory disease, diabetes with complications, dependence on dialysis or end-stage renal disease, liver disease, hematologic conditions, severe obesity (body mass index ≥ 40 kg/m^2^), nursing home residence, and moderate or severe immune compromise. Abbreviations: EMA, European Medicines Agency; FDA, Food and Drug Administration; RSV, respiratory syncytial virus; RSV-LRTD, respiratory-syncytial-virus-associated lower respiratory tract disease.

**Table 2:** Efficacy of the three approved vaccines against RSV-associated LRTD in older adults.

	Vaccine Efficacy (%, CI, Cases in Vaccine Arm/Cases in Placebo Arm)		
Endpoint	RSVPreF3	RSVPreF	mRNA-1345
**Season 1**			
RSV-LRTD *	**82.6%** (96.95% CI, 57.9–94.1) 7/12,466 vs. 40/12,494 [51]	N/A	N/A
RSV-LRTD with ≥2 symptoms	N/A	**65.1%** (95% CI, 35.9–82.0) 15/18,050 vs. 43/18,074 [52]	**78.7%** (95.04% CI, 62.8–87.9) 15/17,561 vs. 70/17,503 [53]
RSV-LRTD with ≥3 symptoms	N/A	**88.9%** (95% CI, 53.7–?) ^ 2/18,050 vs. 18/18,074 [52]	**80.9%** (95.1% CI, 50.1–92.7) 5/17,561 vs. 26/17,503 [53]
Severe RSV-LRTD	**94.1%** ^†^ (95% CI, 62.4–99.9) 1/12,466 vs. 17/12,494 [51]	N/A	**86.7%** ** (95% CI, 41.9–97.0) [53]
RSV-LRTD in participants with ≥1 pre-existing comorbidity of interest	**94.6%** (95% CI, 65.9–99.9) 1/4937 vs. 18/4861 [51]	N/A	N/A
**Season 2**			
RSV-LRTD *	**56.1%** (95% CI, 28.2–74.4) 20/4991 vs. 91/10,031 [51]	N/A	N/A
RSV-LRTD with ≥2 symptoms	N/A	**55.7%** (95% CI, 34.7–70.4) 39/16,164 vs. 88/16,059 [52]	**62.5%** (95.04% CI, 47.7–73.1) 48/18,074 vs. 127/18,010 [53]
RSV-LRTID with ≥3 symptoms	N/A	**77.8%** (95% CI, 51.4–91.1) 8/16,164 vs. 36/16,059 [52]	**61.1%** (95.1% CI, 34.7–76.8) 20/18,074 vs. 51/18,010 [53]
Severe RSV-LRTD	**64.2%** ^†^ (95% CI, 6.19–89.2) 5/4991 vs. 28/10,031 [51]	N/A	**74.6%** ** (95% CI, 50.7–86.9) [53]
RSV-LRTD in participants with ≥1 pre-existing comorbidity of interest	**51.5%** (95% CI, 7.4–76.6) 12/1981 vs. 48/3895 [51]	N/A	N/A
**Season 3**			
RSV-LRTD *	**48.0%** (95% CI, 8.7–72.0) 16/4988 vs. 61/10,031 [51]	N/A	N/A
RSV-LRTD with ≥2 symptoms	N/A	N/A	**50.3%** (95.04% CI, 37.5–60.7) 113/18,181 vs. 225/18,132 [53]
RSV-LRTD with ≥3 symptoms	N/A	N/A	**49.9%** (95.1% CI, 27.8–65.6) 46/18,181 vs. 91/18,132 [53]
Severe RSV-LRTD	**43.3%** ^†^ (95% CI, −45.3–81.3) 6/4988 vs. 21/10,031 [51]	N/A	**56.7%** ** (95% CI, 33.1–72.6) [53]
RSV-LRTD in participants with ≥1 pre-existing comorbidity of interest	**57.8%** (95% CI, 8.0–83.0) 8/2000 vs. 37/3924 [51]	N/A	N/A
**Cumulative**	**Over 3 seasons** with season as covariate		
RSV-LRTD *	**62.9%** (97.5% CI, 46.7–74.8) 48/12,468 vs. 215/12,498 [51]	N/A	N/A
RSV-LRTD with ≥2 symptoms	N/A	N/A	**63.3%** (up to 12 months) (95% CI, 48.7–73.7) 47/18,112 vs. 127/18,045 [54]
RSV-LRTD with ≥3 symptoms	N/A	N/A	**63.0%** (up to 12 months) (95% CI, 37.3–78.2) 19/18,112 vs. 51/18,045 [54]
Severe RSV-LRTD	**67.4%** ^†^ (95% CI, 42.4–82.7) 15/12,468 vs. 75/12,498 [51]	N/A	N/A
RSV-LRTD in participants with ≥1 pre-existing comorbidity of interest	**64.7%** (95% CI, 45.1–78.1) 25/5014 vs. 116/4951 [51]	N/A	**63.4%** (up to 24 months) (95% CI, 45.4–75.5) 33/5393 vs. 88/5276 [53]

* Defined as ≥2 lower respiratory symptoms or signs (including ≥1 lower respiratory sign for ≥24 h), or ≥3 lower respiratory symptoms lasting for ≥24 h [55]. ^ Walsh et al. reported an upper 95% CI of 8.7, but we suspect that this may be a typo [52]. ^†^ Severe RSV-LRTD was determined on the basis of either of two case definitions: on the basis of clinical signs or investigator assessment or on the basis of receipt of supportive therapy [55]. ** RSV-LRTD-associated shortness of breath. Abbreviations: CI: confidence interval; N/A: not available; RSV, respiratory syncytial virus; RSV-LRTD: respiratory-syncytial-virus-associated lower respiratory tract disease.
